# Engineered Lipidic Nanomaterials Inspired by Sphingomyelin Metabolism for Cancer Therapy

**DOI:** 10.3390/molecules28145366

**Published:** 2023-07-12

**Authors:** Han Zhu, Hua-Jie Chen, Hai-Yan Wen, Zhi-Gang Wang, Shu-Lin Liu

**Affiliations:** 1 State Key Laboratory of Medicinal Chemical Biology, Tianjin Key Laboratory of Biosensing and Molecular Recognition, Research Center for Analytical Sciences, College of Chemistry, and School of Medicine, Nankai University, Tianjin 300071, China; 2 Engineering Research Center of Nano-Geomaterials of Ministry of Education, Faculty of Materials Science and Chemistry, China University of Geosciences, Wuhan 430074, China

**Keywords:** cancer therapy, lipidic nanomaterial, sphingomyelin metabolism, tumor progression

## Abstract

Sphingomyelin (SM) and its metabolites are crucial regulators of tumor cell growth, differentiation, senescence, and programmed cell death. With the rise in lipid-based nanomaterials, engineered lipidic nanomaterials inspired by SM metabolism, corresponding lipid targeting, and signaling activation have made fascinating advances in cancer therapeutic processes. In this review, we first described the specific pathways of SM metabolism and the roles of their associated bioactive molecules in mediating cell survival or death. We next summarized the advantages and specific applications of SM metabolism-based lipidic nanomaterials in specific cancer therapies. Finally, we discussed the challenges and perspectives of this emerging and promising SM metabolism-based nanomaterials research area.

## 1. Introduction

Human mortality is becoming increasingly dominated by cancer, which has long posed a significant public health concern [1]. According to the latest estimates by the World Health Organization (WHO)’s International Agency for Research on Cancer (IARC), the number of new cancer cases reported worldwide have risen to 19.29 million, with 9.96 million deaths reported by the year of 2020 [2]. For decades, scientists have been seeking effective cancer therapeutic targets and developing innovative cancer treatment strategies to defeat cancer. Over the past few decades, combining nanotechnology, materials science, and biotechnology has accelerated the development of the innovative nanomedicine field for cancer treatment [3]. Due to the unique appeal of nanomaterials for drug delivery, cancer diagnosis and imaging, and immune vaccine development, interest in applying nanotechnology to cancer treatment has grown, and a considerable level of technical success has been achieved [4,5]. Notable examples include small superparamagnetic iron oxide nanoparticles, which have been widely used as magnetic resonance imaging (MRI) contrast agents, and the successful introduction of the lipid nanoparticle COVID-19 mRNA vaccine [6,7]. Lipidic nanomaterials are nanoparticles consisting of lipid-like substances, with their diameters typically being between 10–200 nm [8]. As one of the most popular platforms for cancer treatment, they have demonstrated tremendous therapeutic potential across a wide range of treatment modalities. The natural origin of lipids ensures an ultra-high biocompatibility and degradability, making them superior to other materials like polymers due to their low probability of causing lipid-induced toxicity [8]. Moreover, they also exhibit attractive multifunctional properties, including an encapsulation capability for either hydrophilic or hydrophobic therapeutics, as well as surface properties, such as charge and targeting ligand modifications, that can be easily modified by altering the lipid composition or by attaching antibodies [9,10]. This flexibility allows for precise drug delivery and targeted therapy, making lipidic nanomaterials promising candidates for innovative cancer therapeutic strategies.

The emergence of lipidomic and lipid quantification in recent years has provided large-scale qualitative and quantitative studies of lipid compounds in organisms, providing insights into the functions and changes of lipid compounds under physiopathological conditions, and has also significantly contributed to the development of lipidic nanomaterials [11,12,13]. There is growing evidence in that when SM levels are expressed abnormally, it increases the cancer risk [14,15]. Among them, biological studies on the metabolism and function of sphingomyelin (SM) have revealed that effector molecules in the SM metabolic pathway contribute significantly to cell signaling, especially in regulating tumor cell growth, differentiation, senescence, and survival [16,17,18]. SM is integral for the membrane, and is an essential structural molecule in the cell membrane. Related enzymes, such as sphingomyelinase (SMase) and sphingomyelin synthase (SMS), strongly regulate its abundance [19]. A critical role for SM content and metabolic enzyme activity in signaling pathway transduction and therapy must be considered. Furthermore, SM metabolites have diverse biological functions across various cancerous processes [20]. For example, ceramide (Cer), produced by the SMase-mediated hydrolysis of SM, is closely associated with cell death, senescence, and cellular arrest [21,22]. Upon further metabolism, Cer can be converted to a pro-survival metabolite called sphingosine-1-phosphate (S1P), which is an anti-apoptotic molecule [23,24]. Thus, the dynamic transformation processes associated with SM metabolism are critical for mediating the fate of cancer cells.

Currently, an increasing number of investigators are focusing on the design of lipidic nanomaterials based on SM metabolism, initiating preclinical trials for different types of tumors to evaluate the potential application of therapeutic strategies utilizing SM, Cer, and S1P [25,26]. In this review, we first describe the distribution and function of SM metabolism-related molecules. We then present the latest evidence regarding SM metabolism in tumor formation and progression, which has profound implications for understanding various aspects of cancer biology and therapeutics concerning SM. We next summarize the recent advances in emerging lipidic nanomaterials involving SM metabolism that have been attempted and established for their use in tumors. We conclude with an outlook on the possibilities and challenges of utilizing SM as a therapeutic target.

## 2. Synthesis and Metabolism of SM

### 2.1. Structure and Distribution of SM

**Figure 1:**
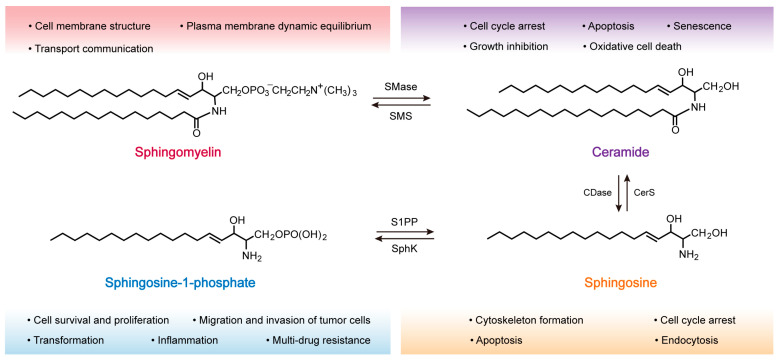
Chemical structure formula of sphingomyelin and its metabolites. The biological roles of the relevant signaling molecules are labeled under different colored fonts. Sphingomyelinase (SMase), sphingomyelin synthase (SMS), ceramidase (CDase), ceramide synthase (CerS), sphingosine kinase (SphK), and sphingosine-1-phosphate phosphatase (S1PP).

**Figure 2:**
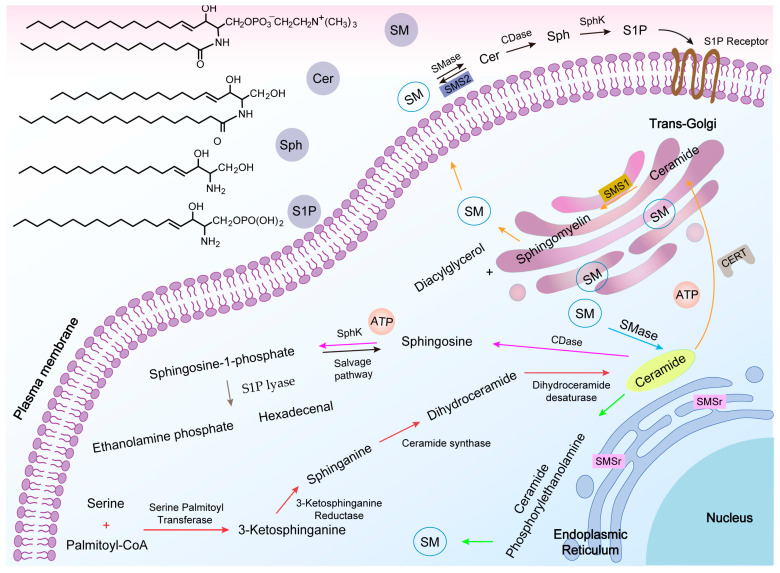
Sphingomyelin-related metabolic pathways. The relevant chemical structures are shown (above left). Ceramide (Cer) de novo synthesis starts from the endoplasmic reticulum (ER), and once synthesized, can be transferred to the trans-Golgi via the ceramide transfer protein (CERT), or be reorganized via a salvage pathway (red arrow). The action of sphingomyelin synthase 1 (SMS1) on Cer leads to the production of sphingomyelin (SM) (orange arrow). In response to the stimulus of apoptosis, phospholipid disorganization moves the isolated SM from the outer leaflet to the cytoplasmic side of the plasma membrane so that sphingomyelinase (SMase) can act on them to produce apoptotic Cer, with the opposite process occurring via sphingomyelin synthase (SMS) (black arrow). Second, Cer can be produced via the SMase (blue arrow). SM can also be produced by converting Cer via sphingomyelin synthase-associated protein (SMSr) (green arrow). Sphingosine (Sph) is produced by the hydrolysis of Cer by ceramidase (CDase), which is then phosphorylated by sphingosine kinase (SphK) to produce sphingosine-1-phosphate (S1P) (pink arrow). The presence of S1P lyase (SPL) also allows S1P to produce ethanolamine phosphate and hexadecenal (brown arrow).

In the 1880s, Johann Ludwig Wilhelm Thudichum [27] discovered and named the first sphingolipid SM, which is a phospholipid that is mainly found in vertebrates, accounting for 2% to 15% of total phospholipids, respectively [27]. Each molecule of SM is composed of a sphingosine (Sph) molecule, a fatty acid, and phosphorylcholine polar head group, and is characterized by a hydrophilic head and a hydrophobic tail made of two fatty acid chains, allowing them to form a lipid bilayer, the chemical structure of which is shown in Figure 1 (top left) [28,29]. The hydrophobic tail of SM is typically 16 to 24 carbons in length. At the same time, the sphingoid backbone can be either dihydrosphingosine, sphingosine, or 4-hydroxysphinganine, thus conferring heterogeneity to the SM molecule [30]. Various studies have shown that SM is present in most intracellular compartments, including the Golgi apparatus (which produces most of the SM molecules) and the plasma membrane (PM) (Figure 2) [16]. SM is also highly enriched in the outer leaflet of the PM and is the major component of the PM [31]. Exceptionally, erythrocytes and neural tissue have a higher SM content [20]. It has been estimated that the proportion of SM in the PM of fibroblasts can account for 40% to 90% of the total SM in the cell, respectively [32].

### 2.2. SM Synthesis

SM is synthesized by two major isoforms of SMS, SMS1 (located in the trans-Golgi) and SMS2 (located in the trans-Golgi and PM) [33]. SMS2 in the PM is the only enzyme capable of synthesizing SM, and may have the unique function of directly maintaining SM content in the PM. It is unknown where the primary site of SM synthesis is located, but it occurs either on the luminal side of the trans-Golgi or in the extracellular leaflet of the PM (orange arrow in Figure 2) [34,35]. Cer is the substrate for this reaction, and this molecule is synthesized in the endoplasmic reticulum (ER). Thus, Cer must be transferred from its site of synthesis to the Golgi via a mediator termed the ceramide transfer protein (CERT) (a non-vesicular mechanism). The transfer of a phosphocholine headgroup from phosphatidylcholine to Cer to generate SM is catalyzed by SMS1, along with the generation of diacylglycerol (DAG) [36]. CERT is a cytoplasmic protein with two unique domains: the START (StAR-related lipid-transfer) domain for Cer recognition and the PH (pleckstrin homology) domain for phosphatidylinositol binding [36,37]. Unlike SMS1, SMS2 is not reliant on CERT-mediated Cer molecules to synthesize SM due to its unique cellular location, and is the only type of SMS present in the PM. Its most prominent mechanism is likely in converting local Cer to SM [38]. Sphingomyelin synthase-related protein (SMSr) is an enzyme present in the ER that is capable of converting Cer to ceramide phosphorylethanolamine (CPE), followed by the head group being exchanged or methylated to produce SM (green arrow in Figure 2) [31,39]. As CPE synthesizes extremely slowly, SM is produced in much lower amounts than when SM is synthesized in the Golgi, and it is commonly believed that SMSr regulates the balance of Cer in the ER to prevent excessive accumulation of Cer.

### 2.3. SM Metabolism

The metabolites of SM include Cer, Sph, and S1P, which are an essential class of biologically active signaling molecules [40]. Cer and Sph are important components of the stress response regulatory system, mediating cell cycle arrest and inducing apoptosis. However, S1P exhibits the opposite effect, promoting cell growth, invasion, and survival [41]. The synthesis and catabolism of these metabolites constitute a dynamic equilibrium relationship that judges the cell fates (e.g., cell proliferation, differentiation, or apoptosis). The regulation of these interconnected cell fates results from interactions and mutual equilibria between regulatory molecules within the cell. This subtle relationship between Cer, Sph, and S1P has been described as a “sphingomyelin rheostat” [21,22].

#### 2.3.1. Ceramide (Cer)

Cer is a family of hydrophobic molecules that is a pivotal center of SM metabolism, containing a fatty acid fraction (C2 to C28) linked to sphingosine or related long-chain bases in its structure. Its biological functions mainly include the induction of apoptosis, regulation of cell differentiation, and immunity [42,43]. The functions performed by Cer differ depending on the accumulation location in cells [44]. For example, Cer produced by SM hydrolysis accumulates in the PM, contributing to forming a larger “ceramide platform” that promotes the aggregation of receptor molecules. This can enhance signaling and lead to the inhibition of cell growth and cell death mediated by stress processes. Enzymes that regulate Cer production, including CerS, SMases, and SphK, are distributed in the mitochondria, PM, or lysosomes, and can affect different cell death mechanisms (apoptosis and necrosis) [45,46,47].

Cer can be produced in three ways; first, through the de novo synthesis pathway. This process is triggered by the formation of 3-ketosphinganine from serine and palmitoyl-CoA through the actions of serine palmitoyl-CoA transferase (SPT) with the participation of NADPH-dependent enzymes, which is then converted to dihydroceramide through the actions of ceramide synthase (CerS), and finally to Cer by dehydrogenation, as indicated by the red arrow in Figure 2 [48]. Cer is then utilized in SM synthesis by SMS activity [49]. This pathway occurs in the ER and is characterized by its Golgi-dependent nature. After Cer is synthesized, CERT promotes Cer transport to the Golgi for further SM synthesis, which is ATP-dependent, as described previously. There is also a vesicle route for Cer transport to the Golgi. Unlike the route described above, this can synthesize glycosyl ceramides, and is not described in detail in this paper [50]. Second, Cer can be produced through the sphingomyelinase pathway (blue arrow in Figure 2). This pathway’s exact process depends upon which SMase type is activated. The evidence suggests that a variety of stimuli, including anticancer drugs, radiation, and inflammatory cytokines, can activate SMase to catalyze the hydrolysis of SM into Cer through the removal of the phosphorylcholine groups [51,52]. The specific SMases involved in this reaction are classified as either neutral, acid, or alkaline sphingomyelinases by their optimal pH. Neutral sphingomyelinase (n-SMase) is present in the PM, cytoplasm, and nucleus, and is associated with cell death mediated by hypoxia, nutrient starvation, and chemotherapeutic drug stimulation [53,54,55]. n-SMase, a central responder to cell death under cytokine activity and stress, can be activated by stimuli such as interleukin (IL)-1β, tumor necrosis factor (TNF)-α, and radiation, but is inhibited by glutathione (GSH), thereby protecting the cell [52,56]. Acid sphingomyelinase (a-SMase) is a lysosomal enzyme that has been highly associated with Niemann–Pick disease, and current data indicates that radiation can act directly on the PM, leading to the activation of a-SMase, mediating Cer production, and initiating the apoptosis reaction [57]. Alkaline sphingomyelinase (alk-SMase) hydrolyzes SM in the intestinal lumen and mucosa and is present in epithelial cells [58]. Recent studies have found low alk-SMase expression levels in colon cancer tissues, and that the production of Cer by this enzyme in the intestinal system may have implications for gastrointestinal tumor biology [59,60]. Third, Cer can be produced by the salvage pathway. SM formed by the degradation of gangliosides and complex sphingolipids is converted back to Cer by ceramidase (CDase) without going through the formation of dihydroceramide [61].

#### 2.3.2. Sphingosine (Sph)

Sph is an 18-carbon amino alcohol with an unsaturated hydrocarbon chain produced by the hydrolysis of Cer by CDase and is one of the components of the cell membrane. Sph is closely associated with cytoskeleton formation, endocytosis, the cell cycle, and apoptotic processes [62]. For Sph metabolism, S1P is produced by the phosphorylation of Sph by SphK (SphK utilizes ATP to phosphorylate the C-1 hydroxy group of free sphingosine), and S1P can be dephosphorylated back to Sph by lipid phosphatase (pink arrow in Figure 2) [63]. However, S1P can also undergo an irreversible reaction in the presence of S1P lyase (SPL) to produce ethanolamine phosphate and hexadecenal [64].

#### 2.3.3. Sphingosine-1-Phosphate (S1P)

S1P, derived from Cer, a common end product of sphingolipid catabolism, is also thought to be the homeostatic point of Cer in cell fate regulation [65]. Cell proliferation and migration, as well as cell survival, are impacted by S1P through its promotion of the homotypic and heterotypic cell-cell interactions [66,67].

S1P is produced when Sph is catalyzed by SphK, a lipid kinase with two isozymes, SphK1 and SphK2 [65]. Sph is phosphorylated to create S1P by SphK1 via interactions with the calcium myristoyl switch protein 1 (CIB1), while SphK2 produces S1P in cells by activating the endonuclear protein kinase C (PKC) [68,69]. Cells use SPL and S1P phosphatase (S1PP) to regulate the concentrations of S1P to circumvent unacceptable levels of S1P in the cytoplasm [70]. S1P exerts its biological effects under normal conditions, mainly through binding to a group of S1P receptors (S1PR) called G protein-coupled receptors, which have five isoforms: S1P1 to S1P5. These molecules also involve numerous biological processes, such as cell proliferation, neoangiogenesis, endothelial cell chemotaxis, and immune cell trafficking [71,72,73].

## 3. Role of SM in the Development of Tumors

Active molecules of the SM-related pathways play a crucial role in tumorigenesis and progression, and alterations in their related signaling play a vital role in the induction of cancer cell death or survival (Table 1).

### 3.1. Oncogenesis

As SM has become more well-understood, it is clear that anabolic-related pathways are increasingly involved in carcinogenesis. SM readily uses its groups to interact with its neighboring molecules and surrounding water molecules to form intermolecular hydrogen bonds, which may enable it to distinguish itself from other lipids [97]. For example, glycerolipids, the major lipid in animal cells, do not have the ability to form interlipid hydrogen bonds [98]. Once this hydrogen bonding network increases significantly, meaning the SM content of the membrane layer increases substantially, and leads to an increased membrane stiffness, reduced fluidity and permeability, inhibition of normal intercellular contacts, and impairment of the metabolic pathways and intercellular and intracellular signaling. This allows intercellular signal transduction, reduced expression of surface molecules, and cell spreading [99,100,101]. From the current study, changes in the SM levels in several types of tumor cells have demonstrated that SM can contribute to the tumorigenic link. For instance, the amount of SM in the extracellular PM lobules is significantly increased in human prostate adenocarcinoma, breast cancer, colon cancer, prostate cancer, kidney cancer, and liver cancer, and the associated metabolic enzymes are also altered, such as SMase and SMS [14,15]. The essential product of SM metabolism, Cer, may be a critical link in cancer progression [102]. Reduced SM metabolism leads to a lower Cer production, as Cer can promote apoptosis, and inhibit cell proliferation and migration [103]. In SM metabolism, the pro-survival and neovascularization observed by the receptor activation of the SM metabolite S1P may be a signaling pathway contributing to tumorigenesis and progression [104].

### 3.2. Proliferation and Metastasis

After tumorigenesis, a sustained increase in the SM content in cell membranes can interfere with normal intercellular communication. Tumor cells use SM-rich PM as a cover to mask their tracks using a tight hydrogen barrier formed by the interaction of SM with its surrounding molecules to ensure that cancer cells cloak their tracks in the vasculature from immune cells and provide shelter for their growth, leading to the formation of distal metastasis [55,97]. The available evidence in the literature suggests that SM, the active component in the extracellular membrane vesicles of tumor cells, may support tumor growth and metastasis by facilitating endothelial cell migration, invasion, and angiogenesis [105]. An early study showed that increased SM synthesis was consistent with decreased Cer levels, suggesting a pro-proliferative function of SM [106]. CDase is overexpressed in hepatocellular carcinoma cell lines and mediates Cer hydrolysis to accelerate cell proliferation [107]. The proliferation and invasiveness of breast cancer are attributed to the inhibition of apoptosis through ceramide-related pathways [108].

Researchers have demonstrated that variations in SMS expression and activity mediate critical steps in tumor proliferation but may be limited by specific environments. For example, researchers used mouse embryonic fibroblasts (MEFs) to demonstrate that the expression of SMS1 or SMS2 enhances proliferation in response to the essential amino acids and growth factors [109]. Increased and abnormal SMS expression levels in ovarian and breast cancers may contribute to tumor metastasis [90,110]. Disrupting the mitochondrial and lipid metabolic pathways by increasing the cellular oxidative damage was shown to prevent ovarian cancer cells’ continued survival and metastasis. This was specifically achieved by depleting SMS2 [111]. One report showed that SMS2-deficient mice exhibit inhibited EL4 cell invasion into the liver with an increased cell survival [112]. The malignant and local invasion of M2 macrophages in the tumor indicates a poor prognosis for patients with triple-negative breast cancer (TNBC), which can be ameliorated by SMS2 inhibition or knockout in TNBC mouse models [113]. These experiments suggest that SMS2 may be a therapeutic target for delaying tumor invasion. S1PR may be a target for preventing tumor angiogenesis, another critical factor in cancer progression. S1P has also been shown to affect epithelial cancer cell migration and contribute to metastatic potential. Chongsathidki et al. identified a brain-specific tumor escape immunosurveillance mechanism in glioblastoma. The prohibition of S1PR internalization licenses previously ineffective T cell activation therapies in brain cancer glioblastoma (GBM) [114]. S1PR2 induces the migration and invasion of HeLa cells [59]. n-SMase2, encoded by SMPD3, catalyzes SM catabolism to produce the anti-tumor metabolite Cer, and is associated with early postoperative recurrence of hepatocellular carcinoma [115]. The hypermethylation or low expression of n-SMase2 is a common event in oral squamous cell carcinoma and renal cell carcinoma, and is an index of tumor cell diffusion, malignancy degree, and early recurrence [116,117]. Immune escape, frequently occurring in human metastatic melanoma, may also be associated with this enzyme downregulation [115].

### 3.3. Multidrug Resistance

Multidrug resistance is a phenomenon in which malignant tumor cells are exposed to one anticancer drug and subsequently become resistant to multiple other anticancer drugs with different structures and mechanisms of action [118]. This phenomenon is frequently observed in recurrent cancers and greatly limits the available treatment options. The principal mechanism by which SM leads to cancer drug resistance is that high levels of SM in the PMs of cancer cells can reduce the influx of anticancer drugs through nanoparticle-based delivery systems, allowing drug sequestration in intracellular vesicles [119]. SM levels can be used to determine the degree of the intake of the anti-pancreatic cancer drug gemcitabine [120]. Regulation of the SM metabolic pathways may be a way to improve the sensitivity of certain tumor chemotherapies by enhancing the apoptotic response. Recent studies have shown that breast cancer stem cells are more resistant to serum deprivation and Cer-induced apoptosis compared to non-stem cells [121]. This feature is from the unique overexpression of SMS1 in stem cells and is likely to be specifically attributed to reduced Cer levels. The role of SMase is crucial for chemotherapy and radiotherapy efficacy. a-SMase-deficient human lymphoblastoid cells and mouse specimens failed to induce apoptosis after being treated with ionizing radiation, as previously shown by Santana et al. [57]. A strong resistance to cisplatin has been observed in melanoma cells with low expression levels of a-SMase, and many studies have also linked this enzyme to resistance in glioblastoma, colon, ovarian, and non-small cell lung cancers, most likely caused from low levels of Cer [95,122,123,124,125]. The slightly acidic environment of certain solid tumors may also lead to increased a-SMase activity, inducing Cer production as a result, and thus leading to cell death [126].

## 4. SM Metabolism-Based Lipidic Nanomaterials for Cancer Therapy

### 4.1. SM-Based Lipidic Nanomaterials for Cancer Therapy

**Figure 3:**
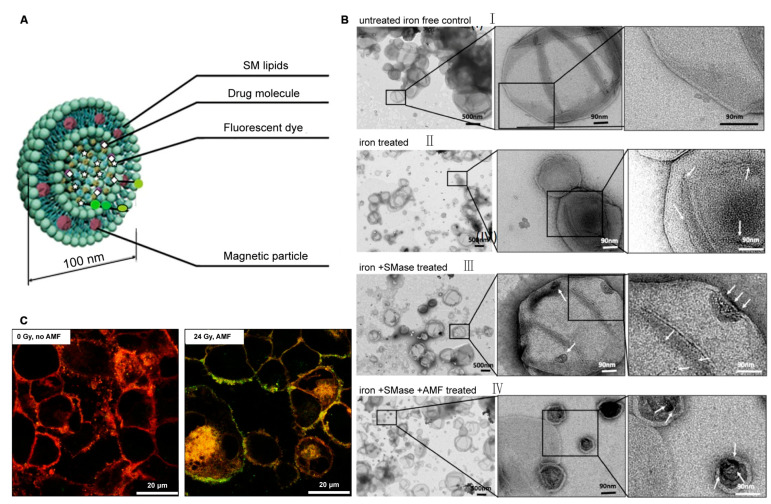
Magnetoenzymatic SM liposomes. (**A**) Schematic illustration of SM liposomes containing Fe_3_O_4_-nanoparticles embedded in the lipid membrane and a payload of imaging and/or drug molecules inside the liposome. (**B**) TEM image of SM liposomes with different treatments. White arrows represent individual iron particles and large domain-like clusters of iron particles. (**C**) Cellular interaction of SCC cells with the fluorescently (TeXas-Red PE) labeled SM liposomes loaded with ferric nanoparticles and calcein. Scale bar: 20 μm. Adapted with permission from ref. [132]. Copyright 2020, MDPI.

Various chemotherapeutic agents, small molecule inhibitors, and microRNAs (miRNAs) have all been used for cancer treatment in the clinic, but often have a poor therapeutic efficacy due to their low in vivo utilization and off-target [127]. To improve their clinical translation, various nanocarriers have been designed to achieve precise targeting and low side effects, enhanced permeability, and retention effects [128]. SM nanosystems have been shown to be promising carriers for effectively delivering anticancer drugs. Recently, Wang et al. [129] developed an SM-derived camptothecin (CPT) (SM-CPT) liposome nanotherapeutic platform, in which the hydroxyl group of SM is partially suffixed with CPT in self-assembly, driven by the amphiphilic nature of SM in aqueous media. This enhances lactone stability and triggers a cytotoxic T lymphocyte (CTL) response to activating anti-tumor immunity. Coupling this with a PD-L1/PD-1 blockade eradicated more than 80% of MC38 colon tumors. Based on the excellent ability of SM nanosystems to bind to different types of drugs, Bouzo et al. [130] proposed the chemical modification of the natural ligands of guanylyl cyclase (GCC) receptors expressed in metastatic colorectal cancer tumors, which were then anchored to SM nanosystems, and finally loaded with the anticancer drug etoposide (Etp) for combination therapy, a strategy that has previously shown strong potential for the treatment of metastatic colorectal cancer cells. Medina et al. [131] increased the homing ability of pancreatic ductal adenocarcinoma (PDAC) tumors for pancreatic tumor imaging by encapsulating iron nanoparticles and the indocyanine green (ICG) in liposomes composed of cationic SM, while embedding RA-96 Fab fragments at the surface of the liposomal nanoparticles. This may be a suitable drug delivery tool for enhancing positive therapeutic outcomes in PDAC patients. Another novel active release system is the encapsulation of cisplatin and ICG in magnetic SM liposomes for their release in response to stress cellular SMase, as shown in Figure 3A. Individual iron particles and large clusters of iron particle structural domains can be observed in TEM images upon iron and SMase treatment and are clearly distinguishable from the untreated control liposomes (Figure 3B). Intracellular release studies were subsequently performed with Texas red membrane-labeled liposomes loaded with calreticulin, and CLSM images demonstrated a high diffusion of calreticulin under alternating magnetic field (AMF) treatment (Figure 3C). This design allowed the system to only open at the appropriate time within the lesion, showing an improved therapeutic efficacy in murine squamous cell carcinoma tumors [132]. RNA-based gene therapy is a prospective option in the fight against cancer, but the efficiency of biological delivery requires further optimization. Farimah et al. [133] developed a gene therapy nanodrug capable of interfering with tumor growth and migration using a biocompatible SM nanosystem to effectively deliver encoded *TP53TG1* plasmids to HCT-116 colorectal cells as treatment. Another SM-based nanosystem (SNs) incorporating a cationic lipid stearylamine (ST) has been developed that is able to support miRNA conjugation by establishing electrostatic interactions (SNs–ST) [134].

**Figure 4:**
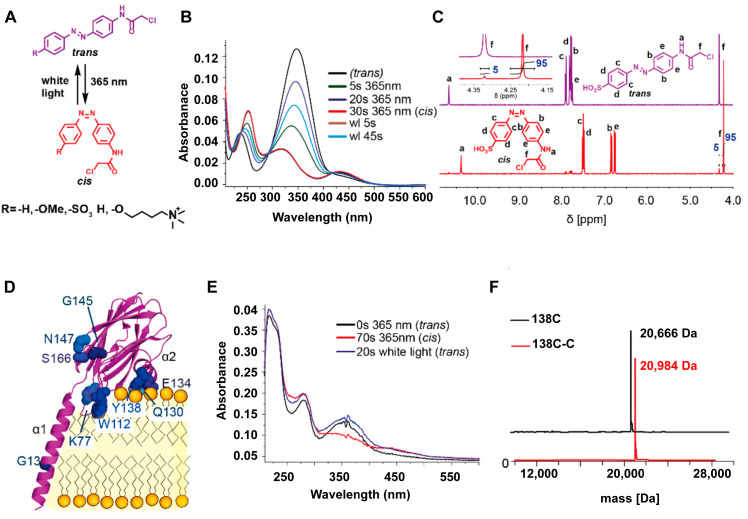
Engineering a light-activated toxin. (**A**) Chemical structures of the azobenzene switch molecules and their activation with light. (**B**) UV–vis spectra of the switch before, during irradiation at 365 nm, and after reaching the PSS state, as well as back-switching with white light (>450 nm). (**C**) ^1^H NMR spectra of C in dimethyl sulfoxide-d_6_ (DMSO-d_6_) (*trans*, purple spectrum) and after irradiation at 365 nm (*cis*, red spectrum). The azobenzene was converted from the *trans* to the *cis* state, and a new signal appeared in the ^1^H NMR spectrum as peak F. (**D**) Cartoon representation of FraC bound to lipids, showing the amino acids substituted with cysteine. (**E**) UV−vis spectra of FraC-Y138C-C before irradiation, during irradiation at 365 nm, and after irradiation with white light. (**F**) ESI-MS spectra of FraC 138C and FraC-Y138C-C. Adapted with permission from ref. [135]. Copyright 2019, American Chemical Society.

These designs were later developed to utilize the unique structure of SM for cancer treatment. Mutter et al. [135] exploited the structural changes that occur when the toxin Fragaceatoxin C (FraC) binds to SM-rich cells (FraC (purple) bound to lipids (yellow) is shown in Figure 4D), leading to nanopore assembly on the cell membrane and subsequent cell death. By attaching the azobenzene switch at multiple locations near the SM-binding pocket of FraC, Figure 4A demonstrates the photoactivation properties of the azobenzene switch. The successful demonstration of the switching properties of the synthesized compounds and the typical features of the azobenzene fraction were investigated using ^1^H NMR spectra, ESI–MS spectrometry, and UV–vis spectroscopy (Figure 4B,C,E,F). With light reversibly controlling the opening and closing of the nanopore, FraC can be activated to mediate the killing of cancer cells, and then inactivated as it diffuses away from the target. When the azobenzene is in the trans-state, FraC cannot bind to the cell surface due to the charged group on the para position that limits the affinity to the hydrophobic bilayer. In contrast, under light irradiation, the azobenzene can be reversibly switched to the cis-state, permitting binding to the cell surface and stabilization by the cis azobenzene to form the pore structure. This approach could be used for future chemotherapy [135]. The synthetically similar product of the glycerophospholipids of the glycerol backbone are known as anti-tumor compounds, while sphingolipid analogs can trigger cytoprotective activity by inhibiting sphingomyelinase. One study suggests that the structural similarity between the 3-deoxy sphingolipids and glycerophospholipids may indicate that the 3-deoxy sphingolipid analogs could be strong candidates for cell-killing drugs. Thus, Hassan et al. [136] comprehensively investigated novel pyrrolidine-based 3-deoxysphingosine analogs that could be used to develop new cancer therapies.

### 4.2. Cer-Based Lipidic Nanomaterials for Cancer Therapy

**Figure 5:**
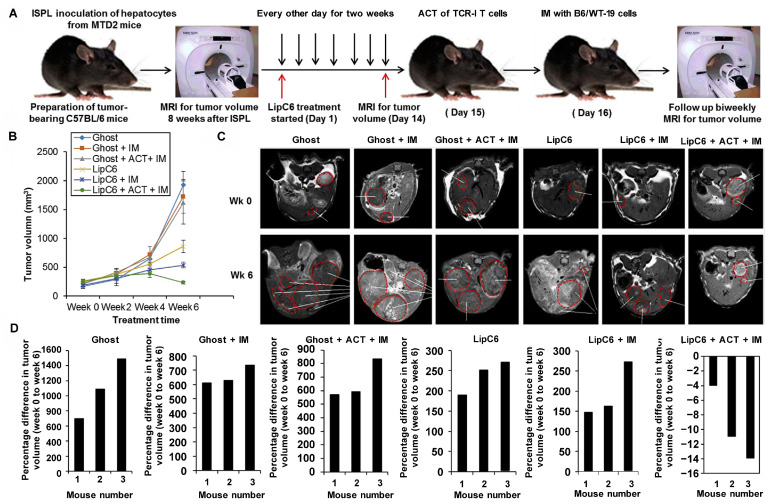
LipC6 injection in combination with tumor antigen-specific (TAS) adoptive cell transfer (ACT) and immunization blocks tumor growth and effectively abolishes established hepatocellular cancer (HCC) tumors. (**A**) Experimental design for therapeutic trial. (**B**) Mean tumor volume in mice. (**C**) Representative images of MRI scans to detect tumors from the start to the endpoints. Tumors are circled in red dotted lines in the images. (**D**) Waterfall plots showing the change in the tumor volume at the experimental endpoint. Adapted with permission from ref. [144]. Copyright 2018, AGA Institute Terms and Conditions.

Cer-mediated anticancer effects have been demonstrated in various cancers (pancreatic, breast, and gastric), affecting T-cell signaling and inducing apoptosis in cancer cells [137,138,139]. However, the cellular impermeability and hydrophobicity of Cer greatly limits its practical application. Several delivery vehicles have been developed to increase Cer delivery and enhance its pharmacological effects, such as calcium phosphate nanocomposite particles, nanoemulsions, liposomes, and others [140,141,142,143]. Li et al. [144] developed C6-Cer-containing nanoliposomes (LipC6) to overcome this limitation for improving the efficacy of immunotherapy in patients with liver cancer. The treatment regimen for the hepatocellular cancer (HCC) model mice is shown in Figure 5A. It was shown that LipC6 slowed tumor growth by reducing tumor cell proliferation, and the triple therapy combined with Lipc6 eventually led to tumor regression (Figure 5B–D). It also induced the differentiation of tumor-associated macrophages (TAMs) into the tumor-killing M1 phenotype following tail vein injection (35 mg/kg for two weeks). This reduced immunosuppression and increased CD^8+^ T cell activity, thus supporting an anti-tumor capacity. Curcumin must be used at relatively high doses (30–100 μM) to kill melanoma cell lines, whereas low doses of curcumin (1–10 μM) in the presence of C6-Cer can either have the same or an even more potent effect on melanoma cell death/apoptosis [145]. Another report demonstrated that LipC6, in combination with gemcitabine or glucosylceramide synthase inhibitors, were effective anti-pancreatic cancer therapeutic agents. It was demonstrated that PANC-1 tumor growth was almost entirely blocked by tail vein injections of 18 mg/kg C6-ceramide [146]. Kester et al. [140] verified that Cer-encapsulated calcium phosphate nanocomposite particles could induce cell death in drug-resistant breast cancer and melanoma cells in vitro, making them sensitive to drug treatment. Sanjay et al. [147] proposed that the co-delivery of doxorubicin, combretastatin-A4, and dexamethasone with a self-assembled lithophane-dipeptide-derived hydrogel (TRI-Gel) induced sphingolipid gene transcriptome-wide variable splicing, increased the level of Cer responsible for apoptosis, and decreased glucosyl Cer to overcome chemoresistance. Medatwal et al. [148] also demonstrated that the combined action of hydrogel-mediated celastrol and doxorubicin resulted in changes in sphingolipid expression and increased ceramide levels, which induced effective tumor regression as a result. The highly brain-permeable drug fluoxetine effectively inhibited the activity of SMPD1, which regulates the transformation of SM to Cer, effectively treating GBM [149].

### 4.3. S1P-Based Lipidic Nanomaterials for Cancer Therapy

**Figure 6:**
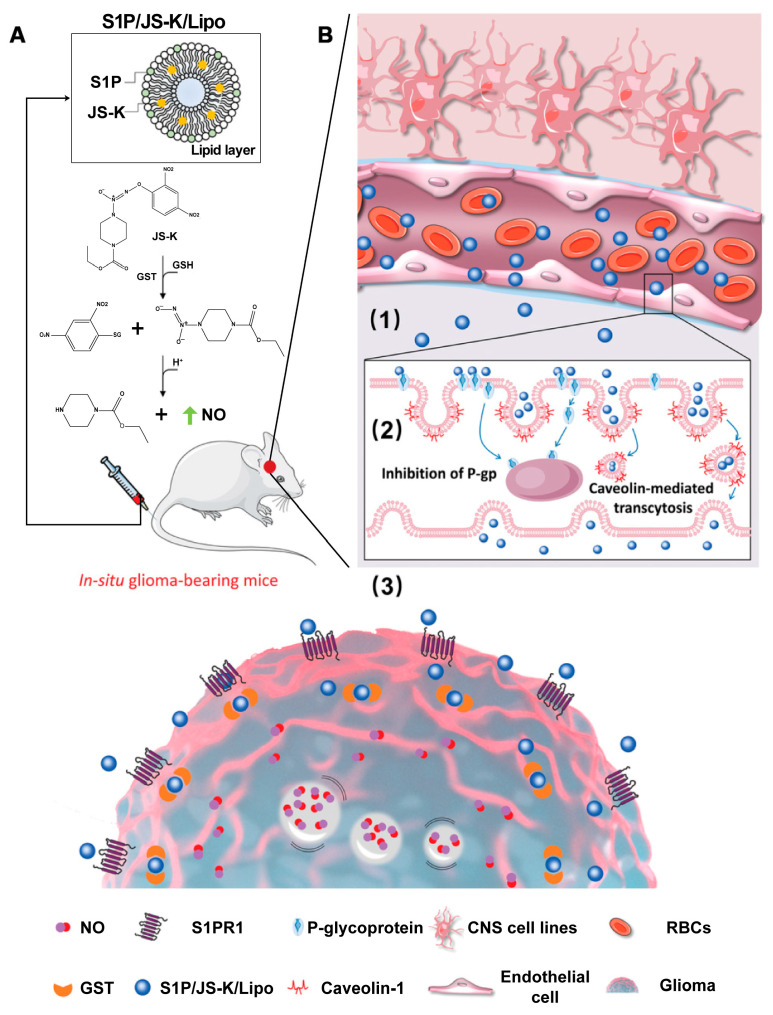
Design features and the proposed mechanism of action of S1P/JS-K liposome targeting of brain glioma tumors. (**A**) Schematic illustration of the preparation of S1P/JS-K/Lipo. (**B**) Schematic indicating the anticipated sequential targeting of S1P/JS-K/Lipo to the BBTB and to brain glioma tumors in mice: (1) S1P/JS-K/Lipo encounters the surface of tumor endothelial cells based on the specific S1P interaction with S1PRs on the endothelial cells. S1P/JS-K/Lipo accumulates at the glioma tumor region; (2) S1P/JS-K/Lipo transmigrates across the BBTB via caveolae-mediated transendothelial transcytosis with inhibition of the P-glycoprotein (P-gp); and the (3) S1P/JS-K/Lipo penetrates glioma tumor cells. Adapted with permission from ref. [150]. Copyright 2021, Wiley-VCH GmbH.

S1P is a lipid member of the SM metabolic pathway that facilitates tumor progression. To address the lack of effective targets for GBM and the low accumulation of drugs in the diseased area from the blood–brain-tumor barrier (BBTB), Liu et al. [150] developed a liposome-based GBM drug delivery system, S1P/JS-K/Lipo, which utilized S1P as its active lipid ligand. This enabled the active penetration of nitric oxide (NO) prodrugs (JS-K, O2-(2,4-dinitrophenyl) 1-[(4-ethoxycarbonyl) piperazin-1-yl] diazen-1-ium-1,2-diolate) into the BBTB to produce tiny NO bubbles for killing tumor cells. When S1P/JS-K/Lipo (equivalent to 2 × 10^−3^ mg kg^−1^ body weight of S1P and 1 mg kg^−1^ body weight of JS-K) was intravenously administered to mice, significant inhibitions of U87MG-RFP-tumor growth was observed. The system was also demonstrated to adequately interact specifically with the highly expressed S1PRs on GBM cells for an efficient targeted delivery, as shown in Figure 6. The FDA-approved drug FTY720 (fingolimod) impairs acid-induced osteosarcoma cell survival and migration by reducing S1P levels. It is currently in the experimental phase as an immunomodulatory agent, and may be useful in designing nanotherapeutic agents [151]. To date, various types of S1P signaling modulators have been developed, including S1P agonists, S1P antagonists, and SphK inhibitors [152]. Their positive impact on cancer therapy (including, but not limited to increased sensitivity to chemotherapy and improved oncologic outcomes) has been described in the literature [153,154,155]. Among them includes the sphingolipid phosphatase SGPP1, an antagonist of S1P signaling which can improve the radiosensitivity of miR-95 overexpressed mice [156]. Additionally, restraining S1P synthesis by inhibiting SphK activity can be used to heighten the response of cancer cells to cytotoxic treatments, and these studies will be a strong basis for future nanomedicine design [157,158].

Finally, recent lipidic nanomaterials designed based on SM metabolism for cancer therapy were summarized, and their related parameters are listed in Table 2.

## 5. Conclusions and Perspective

SM is a vital class of membrane lipid that helps in maintaining the cellular PM’s structure, dynamic homeostasis, and transport communication. Although the biological activity of SM has not been well studied with tumors, various studies have shown that lipid molecules associated with SM metabolism play key roles in cancer cell fate. These associated signaling pathways and metabolites may be important indicators of tumor progression. Despite the proliferation of nanotechnologies, lipidic nanomaterials remain as one of the most promising platforms for in vivo applications. Their natural biodegradability, biocompatibility, and other easily scalable characteristics make them safe for preclinical use and easy to mass produce for cancer treatment. Therefore, the design of SM metabolism-based lipidic nanomaterials may be of interest for tumor therapy. In this article, we reviewed the rationale and design of several innovative lipidic nanomaterials based on SM metabolism and described the unique anti-tumor activities they have shown in in vitro and in vivo experiments.

However, which SM metabolite-based design is the most valuable direction for cancer treatment has not been determined. As apoptosis is a crucial aspect of evaluating the tumor efficacy, and Cer and S1P are key signaling molecules that regulate apoptosis, controlling SM metabolism levels to increase the apoptotic potential of tumor cells may be a promising approach. SM-based lipidic nanomaterials are often effective carriers for chemotherapeutic drug delivery. Artificially fabricated liposomes can hold appreciable amounts of cargo and easily reach where they want to go through simple antibody modifications on their surface. However, bio-delivery efficiency needs to be further optimized by systematically modulating the lipid composition. Furthermore, the unique structure of SM can be used to design personalized response systems to selectively kill cancer cells. The anticancer effect of Cer has been well established, and C6-Cer combination drug therapy can lead to the effective apoptosis of cancer cells or even stronger. Tumor regression can also be induced indirectly by small molecule drugs interfering with the level of intracellular Cer production. However, Cer-based lipidic nanomaterials need to address the in vivo-related biosafety issues, as it is inherently cell-killing. To date, various signal modulators of S1P have been developed, but little is known regarding how they can be designed into nanomaterials that can be utilized to reduce their role in promoting tumor progression, which must be focused on in the future.

Moreover, an essential challenge for SM metabolism-based nanomaterials is the design of various components into them in order to give them any desired function, which also poses considerable difficulties for clinical translation and large-scale production, and the slightest mistake can destroy the superior biosafety of lipid nanomaterials and lose their natural advantages. To achieve this goal, we must better understand the biology of SM in human cancer and how SM signaling pathways can be utilized to design personalized oncology therapies. Focusing on exploiting, modulating, or disrupting SM-related signaling would be a unique and novel strategy for treating cancer.

## Figures and Tables

**Table 1:** The role of active molecules in SM-related pathways in tumorigenesis and development.

Active Molecule	Pathway	Function or Activity	Cancer	Refs.
Sphingosine kinase	Akt/NF-κB	Cancer progression and chemoresistance	Colon	[74]
	S1P/S1PR1	Inflammation and angiogenesis	Breast	[75]
	PI3k/Akt/FOXO3a	Apoptosis resistance	Breast	[76]
	S1P/Stat3/AKT	Proliferation	Colon	[77]
	E-cad	Tumorigenesis and metastasis	Breast	[78]
	S1P/ERK/CD44	Chemoresistance	Colon	[79]
	S1P/AKT/GSK-3β	HIF-1α stabilization	Glioblastoma	[80]
Sphingomyelinase		↑ Sensitivity	Glioblastoma	[81]
		↑ Resistance	Melanoma	[82]
		Induce apoptosis	Colon	[83]
		Induce apoptosis and resistance	Ovarian	[84]
Acid ceramidase		↑ Proliferative ↓ Sensitivity	Melanoma	[85,86]
		↓ Sensitivity	Prostate	[87]
		↑ Radioresistant	Glioblastoma	[87]
Sphingosine kinase 2	Mcl-1	↑ Cell survival	Leukemia	[88]
Sphingosine-1-phosphate lyase	p53 and p38	↑ Apoptosis	Colon	[89]
Sphingomyelin synthase	TGF- b1	↑ Migratory, invasion	Breast	[90]
	Overexpression of SMS1	↓ Cell death	Lymphatic	[91]
Sphingomyelinase	CD95	↑ Apoptosis	Lymphatic	[92]
	p53	↑ Apoptosis	Lung	[93]
Sphingosine	Cdk4	↓ Cell proliferation	Intestinal adenoma	[94]
Ceramide	CerS6/C16-ceramide activated	↑ Apoptosis	Lung	[95]
	High cytotoxicity in p53	↑ Apoptosis	Breast	[96]

↑, increase; ↓, decrease.

**Table 2:** SM metabolism-based lipidic nanomaterials for cancer therapy.

Materials	Size (d. nm)	Therapeutics	Cancer Cell Type	Refs.
SM-CSS-CPT	93.1 ± 7.63	Intravenous injection	CT26, b16, mc38	[129]
UroGm-SNs	131 ± 12		SW620	[130]
DOTAP (DSN)	142 ± 2		HCT-116	[133]
PEGylated Cu (DDC)_2_ liposomes	121.5 ± 0.57	Intravenous injection	4T1	[159]
SNs–ST	131 ± 8		SW480	[134]
SNs_PEG	77 ± 3		PANC-1,	[160]
Lipid–porphyrin conjugates	180 ± 10		Kyse-30	[161]
SMLs@PDA	229.5 ± 26.3			[162]
C6-NBD-SM Liposomes	71 ± 3		CCRF-CEM	[163]
CPNPs	20		MCF-7	[142]
C6 ceramide	~80		B16, WM-115	[145]
TRI-Gel		Subcutaneous injection	Lewis lung carcinoma	[147]
Celastrol		Subcutaneous injection	CT26, HCT-8, HCT-116, DLD-1	[148]
LipC6	90	Intra-splenic injections	liver tumors	[144]
Thirty molar percent C6-ceramide in a twelve molar percent pegylated	80	Intraperitoneal injection	SKOV3, TOV112D, A2780, A2780CP, PE01, PE04	[164]
S1P/JS-K/Lipo	189	Intravenous injection	U87MG	[150]
PP2A			A549	[151]
3-[4-(5-aryl-1, 2, 4-oxadiazol-3-yl)-1H-indol-1-yl]propanoic acid series		Intravenous injection	peripheral lymphocyte	[153]

## Data Availability

Not applicable.

## References

[1] Miller K.D., Siegel R.L., Lin C.C., Mariotto A.B., Kramer J.L., Rowland J.H., Stein K.D., Alteri R., Jemal A. (2016). Cancer treatment and survivorship statistics, 2016. CA Cancer J. Clin..

[2] Ferlay J., Colombet M., Soerjomataram I., Parkin D.M., Pineros M., Znaor A., Bray F. (2021). Cancer statistics for the year 2020: An overview. Int. J. Cancer.

[3] Riehemann K., Schneider S.W., Luger T.A., Godin B., Ferrari M., Fuchs H. (2009). Nanomedicine--challenge and perspectives. Angew. Chem. Int. Ed..

[4] Zhou Q., Zhang L., Wu H. (2017). Nanomaterials for cancer therapies. Nanotechnol. Rev..

[5] Sun T., Zhang Y.S., Pang B., Hyun D.C., Yang M., Xia Y. (2014). Engineered nanoparticles for drug delivery in cancer therapy. Angew. Chem. Int. Ed..

[6] Dadfar S.M., Camozzi D., Darguzyte M., Roemhild K., Varvara P., Metselaar J., Banala S., Straub M., Guvener N., Engelmann U. (2020). Size-isolation of superparamagnetic iron oxide nanoparticles improves MRI, MPI and hyperthermia performance. J. Nanobiotechnol..

[7] Li M., Huang Y., Wu J., Li S., Mei M., Chen H., Wang N., Wu W., Zhou B., Tan X. (2023). A PEG-lipid-free COVID-19 mRNA vaccine triggers robust immune responses in mice. Mater. Horiz..

[8] Chaudhari V.S., Murty U.S., Banerjee S. (2020). Lipidic nanomaterials to deliver natural compounds against cancer: A review. Environ. Chem. Lett..

[9] Limongi T., Susa F., Marini M., Allione M., Torre B., Pisano R., di Fabrizio E. (2021). Lipid-based nanovesicular drug delivery systems. Nanomaterials.

[10] Porter C.J., Trevaskis N.L., Charman W.N. (2007). Lipids and lipid-based formulations: Optimizing the oral delivery of lipophilic drugs. Nat. Rev. Drug Discov..

[11] Quehenberger O., Armando A.M., Brown A.H., Milne S.B., Myers D.S., Merrill A.H., Bandyopadhyay S., Jones K.N., Kelly S., Shaner R.L. (2010). Lipidomics reveals a remarkable diversity of lipids in human plasma. J. Lipid Res..

[12] Wolrab D., Jirasko R., Cifkova E., Horing M., Mei D., Chocholouskova M., Peterka O., Idkowiak J., Hrnciarova T., Kuchar L. (2022). Lipidomic profiling of human serum enables detection of pancreatic cancer. Nat. Commun..

[13] Hu Y., Zhang R.Q., Wang Z.G., Liu S.L. (2022). In situ quantification of lipids in live cells by using lipid-binding domain-based biosensors. Bioconjug. Chem..

[14] Radin N.S. (2002). Cancer progression in the kidney and prostate: Vital roles of sphingolipids in chemotherapy. Urology.

[15] Vykoukal J., Fahrmann J.F., Gregg J.R., Tang Z., Basourakos S., Irajizad E., Park S., Yang G., Creighton C.J., Fleury A. (2020). Caveolin-1-mediated sphingolipid oncometabolism underlies a metabolic vulnerability of prostate cancer. Nat. Commun..

[16] D’Angelo G., Moorthi S., Luberto C. (2018). Role and function of sphingomyelin biosynthesis in the development of cancer. Adv. Cancer Res..

[17] Bienias K., Fiedorowicz A., Sadowska A., Prokopiuk S., Car H. (2016). Regulation of sphingomyelin metabolism. Pharmacol. Rep..

[18] Ogretmen B. (2018). Sphingolipid metabolism in cancer signalling and therapy. Nat. Rev. Cancer.

[19] Ogretmen B., Hannun Y.A. (2004). Biologically active sphingolipids in cancer pathogenesis and treatment. Nat. Rev. Cancer.

[20] Modrak D.E., Gold D.V., Goldenberg D.M. (2006). Sphingolipid targets in cancer therapy. Mol. Cancer Ther..

[21] Newton J., Lima S., Maceyka M., Spiegel S. (2015). Revisiting the sphingolipid rheostat: Evolving concepts in cancer therapy. Exp. Cell Res..

[22] Taniguchi M., Okazaki T. (2021). Role of ceramide/sphingomyelin (SM) balance regulated through “SM cycle” in cancer. Cell. Signal..

[23] Cartier A., Hla T. (2019). Sphingosine 1-phosphate: Lipid signaling in pathology and therapy. Science.

[24] Taha T.A., Mullen T.D., Obeid L.M. (2006). A house divided: Ceramide, sphingosine, and sphingosine-1-phosphate in programmed cell death. Biochim. Biophys. Acta.

[25] Kolesnick R. (2002). The therapeutic potential of modulating the ceramide/sphingomyelin pathway. J. Clin. Investig..

[26] Huwiler A., Pfeilschifter J. (2006). Altering the sphingosine-1-phosphate/ceramide balance: A promising approach for tumor therapy. Curr. Pharm. Des..

[27] Merrill A.H., Schmelz E.M., Dillehay D.L., Spiegel S., Shayman J.A., Schroeder J.J., Riley R.T., Voss K.A., Wang E. (1997). Sphingolipids--the enigmatic lipid class: Biochemistry, physiology, and pathophysiology. Toxicol. Appl. Pharmacol..

[28] Filippov A., Oradd G., Lindblom G. (2006). Sphingomyelin structure influences the lateral diffusion and raft formation in lipid bilayers. Biophys. J..

[29] Christie, William W. (2012). Lipids: Their structures and occurrence. Lipid Analysis.

[30] Furland N.E., Zanetti S.R., Oresti G.M., Maldonado E.N., Aveldano M.I. (2007). Ceramides and sphingomyelins with high proportions of very long-chain polyunsaturated fatty acids in mammalian germ cells. J. Biol. Chem..

[31] Slotte J.P. (2013). Biological functions of sphingomyelins. Prog. Lipid Res..

[32] Allan D., Quinn P. (1988). Resynthesis of sphingomyelin from plasma-membrane phosphatidylcholine in BHK cells treated with staphylococcus aureus sphingomyelinase. Biochem. J..

[33] Gault C.R., Obeid L.M., Hannun Y.A. (2010). An overview of sphingolipid metabolism: From synthesis to breakdown. Adv. Exp. Med. Biol..

[34] Futerman A.H., Stieger B., Hubbard A.L., Pagano R.E. (1990). Sphingomyelin synthesis in rat liver occurs predominantly at the cis and medial cisternae of the Golgi apparatus. J. Biol. Chem..

[35] Huitema K., van den Dikkenberg J., Brouwers J.F., Holthuis J.C. (2004). Identification of a family of animal sphingomyelin synthases. EMBO J..

[36] Hanada K., Kumagai K., Yasuda S., Miura Y., Kawano M., Fukasawa M., Nishijima M. (2003). Molecular machinery for non-vesicular trafficking of ceramide. Nature.

[37] Hanada K. (2007). Regulation of CERT-mediated trafficking of ceramide. Chem. Phys. Lipids.

[38] Mitsutake S., Zama K., Yokota H., Yoshida T., Tanaka M., Mitsui M., Ikawa M., Okabe M., Tanaka Y., Yamashita T. (2011). Dynamic modification of sphingomyelin in lipid microdomains controls development of obesity, fatty liver, and type 2 diabetes. J. Biol. Chem..

[39] Vacaru A.M., Tafesse F.G., Ternes P., Kondylis V., Hermansson M., Brouwers J.F., Somerharju P., Rabouille C., Holthuis J.C. (2009). Sphingomyelin synthase-related protein SMSr controls ceramide homeostasis in the ER. J. Cell Biol..

[40] Saddoughi S.A., Song P., Ogretmen B. (2008). Roles of bioactive sphingolipids in cancer biology and therapeutics. Subcell. Biochem..

[41] Haddadi N., Lin Y., Simpson A.M., Nassif N.T., McGowan E.M. (2017). “Dicing and splicing” sphingosine kinase and relevance to cancer. Int. J. Mol. Sci..

[42] Turpin-Nolan S.M., Bruning J.C. (2020). The role of ceramides in metabolic disorders: When size and localization matters. Nat. Rev. Endocrinol..

[43] Hannun Y.A. (1996). Functions of ceramide in coordinating cellular responses to stress. Science.

[44] Testai F.D., Landek M.A., Dawson G. (2004). Regulation of sphingomyelinases in cells of the oligodendrocyte lineage. J. Neurosci. Res..

[45] Hannun Y.A., Obeid L.M. (2017). Sphingolipids and their metabolism in physiology and disease. Nat. Rev. Mol. Cell Biol..

[46] Babiychuk E.B., Monastyrskaya K., Draeger A. (2010). Fluorescent annexin a1 reveals dynamics of ceramide platforms in living cells. Traffic.

[47] Jain A., Beutel O., Ebell K., Korneev S., Holthuis J.C. (2017). Diverting CERT-mediated ceramide transport to mitochondria triggers Bax-dependent apoptosis. J. Cell. Sci..

[48] Chalfant C. (2010). Sphingolipids as Signaling and Regulatory Molecules.

[49] Gomez-Munoz A., Presa N., Gomez-Larrauri A., Rivera I.G., Trueba M., Ordonez M. (2016). Control of inflammatory responses by ceramide, sphingosine 1-phosphate and ceramide 1-phosphate. Prog. Lipid Res..

[50] Hannun Y.A., Obeid L.M. (2008). Principles of bioactive lipid signalling: Lessons from sphingolipids. Nat. Rev. Mol. Cell Biol..

[51] Goi F.M., Alonso A. (2002). Sphingomyelinases: Enzymology and membrane activity. FEBS Lett..

[52] Marchesini N., Hannun Y.A. (2004). Acid and neutral sphingomyelinases: Roles and mechanisms of regulation. Biochem. Cell Biol..

[53] Clarke C.J., Wu B.X., Hannun Y.A. (2011). The neutral sphingomyelinase family: Identifying biochemical connections. Adv. Enzyme Regul..

[54] Takeda Y., Tashima M., Takahashi A., Uchiyama T., Okazaki T. (1999). Ceramide generation in nitric oxide-induced apoptosis. Activation of magnesium-dependent neutral sphingomyelinase via caspase-3. J. Biol. Chem..

[55] Hara S., Nakashima S., Kiyono T., Sawada M., Yoshimura S., Iwama T., Banno Y., Shinoda J., Sakai N. (2004). p53-independent ceramide formation in human glioma cells during gamma-radiation-induced apoptosis. Cell Death Differ..

[56] Schütze S., Machleidt T., Krönke M. (1994). The role of diacylglycerol and ceramide in tumor necrosis factor and interleukin-1 signal transduction. J. Leukoc. Biol..

[57] Santana P., Pena L.A., Haimovitz-Friedman A., Martin S., Green D., McLoughlin M., Cordon-Cardo C., Schuchman E.H., Fuks Z., Kolesnick R. (1996). Acid sphingomyelinase-deficient human lymphoblasts and mice are defective in radiation-induced apoptosis. Cell.

[58] Nilsson A. (1969). The presence of spingomyelin- and ceramide-cleaving enzymes in the small intestinal tract. Biochim. Biophys. Acta.

[59] Duan R.D. (2006). Alkaline sphingomyelinase: An old enzyme with novel implications. BBA-Bioenerg..

[60] Machala M., Prochazkova J., Hofmanova J., Kralikova L., Slavik J., Tylichova Z., Ovesna P., Kozubik A., Vondracek J. (2019). Colon Cancer and Perturbations of the Sphingolipid Metabolism. Int. J. Mol. Sci..

[61] Novgorodov S.A., Wu B.X., Gudz T.I., Bielawski J., Ovchinnikova T.V., Hannun Y.A., Obeid L.M. (2011). Novel pathway of ceramide production in mitochondria: Thioesterase and neutral ceramidase produce ceramide from sphingosine and acyl-CoA. J. Biol. Chem..

[62] Maceyka M., Payne S.G., Milstien S., Spiegel S. (2002). Sphingosine kinase, sphingosine-1-phosphate, and apoptosis. Biochim. Biophys. Acta.

[63] Olivera A., Spiegel S. (2001). Sphingosine kinase: A mediator of vital cellular functions. Prostag. Other Lipid Mediat..

[64] Bandhuvula P., Saba J.D. (2007). Sphingosine-1-phosphate lyase in immunity and cancer: Silencing the siren. Trends Mol. Med..

[65] Hait N.C., Oskeritzian C.A., Paugh S.W., Milstien S., Spiegel S. (2006). Sphingosine kinases, sphingosine 1-phosphate, apoptosis and diseases. Biochim. Biophys. Acta.

[66] Yatomi Y., Ohmori T., Ge R.L., Kazama F., Okamoto H., Sano T., Satoh K., Kume S., Tigyi G., Igarashi Y. (2000). Sphingosine 1-phosphate as a major bioactive lysophospholipid that is released from platelets and interacts with endothelial cells. Blood.

[67] Kluk M.J., Hla T. (2001). Role of the sphingosine 1-phosphate receptor EDG-1 in vascular smooth muscle cell proliferation and migration. Circ. Res..

[68] Adada M.M., Canals D., Jeong N., Kelkar A.D., Hernandez-Corbacho M., Pulkoski-Gross M.J., Donaldson J.C., Hannun Y.A., Obeid L.M. (2015). Intracellular sphingosine kinase 2-derived sphingosine-1-phosphate mediates epidermal growth factor-induced ezrin-radixin-moesin phosphorylation and cancer cell invasion. FASEB J..

[69] Hait N.C., Allegood J., Maceyka M., Strub G.M., Harikumar K.B., Singh S.K., Luo C., Marmorstein R., Kordula T., Milstien S. (2009). Regulation of histone acetylation in the nucleus by sphingosine-1-phosphate. Science.

[70] Alvarez S.E., Milstien S., Spiegel S. (2007). Autocrine and paracrine roles of sphingosine-1-phosphate. Trends Endocrinol. Metab..

[71] Zheng X., Li W., Ren L., Liu J., Pang X., Chen X., Kang D., Wang J., Du G. (2019). The sphingosine kinase-1/sphingosine-1-phosphate axis in cancer: Potential target for anticancer therapy. Pharmacol. Ther..

[72] Camaré C., Trayssac M., Garmy-Susini B., Mucher E., Sabbadini R., Salvayre R., Negre-Salvayre A. (2014). Oxidized LDL-induced angiogenesis involves sphingosine 1-phosphate: Prevention by anti-S1P antibody. Br. J. Pharmacol..

[73] Mukhopadhyay P., Ramanathan R., Takabe K. (2015). S1P promotes breast cancer progression by angiogenesis and lymphangiogenesis. Breast Cancer Manag..

[74] Shamekhi S., Abdolalizadeh J., Ostadrahimi A., Mohammadi S.A., Barzegari A., Lotfi H., Bonabi E., Zarghami N. (2020). Apoptotic effect of saccharomyces cerevisiae on human colon cancer SW480 cells by regulation of Akt/NF-kB signaling pathway. Probiotics Antimicrob. Proteins.

[75] Nagahashi M., Yamada A., Katsuta E., Aoyagi T., Huang W.C., Terracina K.P., Hait N.C., Allegood J.C., Tsuchida J., Yuza K. (2018). Targeting the SphK1/S1P/S1PR1 axis that links obesity, chronic inflammation, and breast cancer metastasis. Cancer Res..

[76] Zhang X., Zhuang T., Liang Z., Li L., Xue M., Liu J., Liang H. (2017). Breast cancer suppression by aplysin is associated with inhibition of PI3K/AKT/FOXO3a pathway. Oncotarget.

[77] Bao Y., Li K., Guo Y., Wang Q., Li Z., Yang Y., Chen Z., Wang J., Zhao W., Zhang H. (2016). Tumor suppressor PRSS8 targets Sphk1/S1P/Stat3/Akt signaling in colorectal cancer. Oncotarget.

[78] Zheng X.D., Zhang Y., Qi X.W., Wang M.H., Sun P., Zhang Y., Jiang J. (2014). Role of Sphk1 in the malignant transformation of breast epithelial cells and breast cancer progression. Indian J. Cancer.

[79] Kawahara S., Otsuji Y., Nakamura M., Murakami M., Murate T., Matsunaga T., Kanoh H., Seishima M., Banno Y., Hara A. (2013). Sphingosine kinase 1 plays a role in the upregulation of CD44 expression through extracellular signal-regulated kinase signaling in human colon cancer cells. Anticancer Drugs.

[80] Ader I., Brizuela L., Bouquerel P., Malavaud B., Cuvillier O. (2008). Sphingosine kinase 1: A new modulator of hypoxia inducible factor 1alpha during hypoxia in human cancer cells. Cancer Res..

[81] Pchejetski D., Golzio M., Bonhoure E., Calvet C., Doumerc N., Garcia V., Mazerolles C., Rischmann P., Teissie J., Malavaud B. (2005). Sphingosine kinase-1 as a chemotherapy sensor in prostate adenocarcinoma cell and mouse models. Cancer Res..

[82] Alshaker H., Wang Q., Kawano Y., Arafat T., Bohler T., Winkler M., Cooper C., Pchejetski D. (2016). Everolimus (RAD001) sensitizes prostate cancer cells to docetaxel by down-regulation of HIF-1alpha and sphingosine kinase 1. Oncotarget.

[83] Sauer L., Nunes J., Salunkhe V., Skalska L., Kohama T., Cuvillier O., Waxman J., Pchejetski D. (2009). Sphingosine kinase 1 inhibition sensitizes hormone-resistant prostate cancer to docetaxel. Int. J. Cancer.

[84] Sukocheva O., Wang L., Verrier E., Vadas M.A., Xia P. (2009). Restoring endocrine response in breast cancer cells by inhibition of the sphingosine kinase-1 signaling pathway. Endocrinology.

[85] Giussani P., Bassi R., Anelli V., Brioschi L., De Zen F., Riccitelli E., Caroli M., Campanella R., Gaini S.M., Viani P. (2012). Glucosylceramide synthase protects glioblastoma cells against autophagic and apoptotic death induced by temozolomide and Paclitaxel. Cancer Investig..

[86] Liu Y.Y., Han T.Y., Giuliano A.E., Cabot M.C. (1999). Expression of glucosylceramide synthase, converting ceramide to glucosylceramide, confers adriamycin resistance in human breast cancer cells. J. Biol. Chem..

[87] Liu Y.Y., Patwardhan G.A., Xie P., Gu X., Giuliano A.E., Cabot M.C. (2011). Glucosylceramide synthase, a factor in modulating drug resistance, is overexpressed in metastatic breast carcinoma. Int. J. Oncol..

[88] LeBlanc F.R., Pearson J.M., Tan S.F., Cheon H., Xing J.C., Dunton W., Feith D.J., Loughran T.P. (2020). Sphingosine kinase-2 is overexpressed in large granular lymphocyte leukaemia and promotes survival through Mcl-1. Br. J. Heaematol..

[89] Oskouian B., Sooriyakumaran P., Borowsky A.D., Crans A., Dillard-Telm L., Tam Y.Y., Bandhuvula P., Saba J.D. (2006). Sphingosine-1-phosphate lyase potentiates apoptosis via p53- and p38-dependent pathways and is down-regulated in colon cancer. Proc. Natl. Acad. Sci. USA.

[90] Zheng K., Chen Z., Feng H., Chen Y., Zhang C., Yu J., Luo Y., Zhao L., Jiang X., Shi F. (2019). Sphingomyelin synthase 2 promotes an aggressive breast cancer phenotype by disrupting the homoeostasis of ceramide and sphingomyelin. Cell Death Dis..

[91] Lafont E., Milhas D., Carpentier S., Garcia V., Jin Z.X., Umehara H., Okazaki T., Schulze-Osthoff K., Levade T., Benoist H. (2010). Caspase-mediated inhibition of sphingomyelin synthesis is involved in FasL-triggered cell death. Cell Death Differ..

[92] Grassme H., Schwarz H., Gulbins E. (2001). Molecular mechanisms of ceramide-mediated CD95 clustering. Biochem. Biophys. Res. Commun..

[93] Eroica S., Susan C.E., Cynthia C., Elroy F. (2014). Characterizing the sphingomyelinase pathway triggered by PRIMA-1 derivatives in lung cancer cells with differing p53 status. Anticancer Res..

[94] Kohno M., Momoi M., Oo M.L., Paik J.H., Lee Y.M., Venkataraman K., Ai Y., Ristimaki A.P., Fyrst H., Sano H. (2006). Intracellular role for sphingosine kinase 1 in intestinal adenoma cell proliferation. Mol. Cell. Biol..

[95] Senkal C.E., Ponnusamy S., Manevich Y., Meyers-Needham M., Saddoughi S.A., Mukhopadyay A., Dent P., Bielawski J., Ogretmen B. (2011). Alteration of ceramide synthase 6/C16-ceramide induces activating transcription factor 6-mediated endoplasmic reticulum (ER) stress and apoptosis via perturbation of cellular Ca2+ and ER/Golgi membrane network. J. Biol. Chem..

[96] Chang W.T., Wu C.Y., Lin Y.C., Wu M.T., Su K.L., Yuan S.S., Wang H.D., Fong Y., Lin Y.H., Chiu C.C. (2019). C(2)-ceramide-induced rb-dominant senescence-like phenotype leads to human breast cancer Mcf-7 escape from p53-dependent cell death. Int. J. Mol. Sci..

[97] Tallima H., Azzazy H.M.E., El Ridi R. (2021). Cell surface sphingomyelin: Key role in cancer initiation, progression, and immune evasion. Lipids Health Dis..

[98] Mombelli E., Morris R., Taylor W., Fraternali F. (2003). Hydrogen-bonding propensities of sphingomyelin in solution and in a bilayer assembly: A molecular dynamics study. Biophys. J..

[99] Barenholz Y., Thompson T.E. (1999). Sphingomyelin: Biophysical aspects. Chem. Phys. Lipids.

[100] Migliardo F., Tallima H., Ridi R.E. (2014). Is there a sphingomyelin-based hydrogen bond barrier at the mammalian host-schistosome parasite interface?. Cell Biochem. Biophys..

[101] Slotte J.P. (2016). The importance of hydrogen bonding in sphingomyelin’s membrane interactions with co-lipids. Biochim. Biophys. Acta.

[102] Li Z., Zhang L., Liu D., Wang C. (2021). Ceramide glycosylation and related enzymes in cancer signaling and therapy. Biomed. Pharmacother..

[103] Sheridan M., Ogretmen B. (2021). The role of ceramide metabolism and signaling in the regulation of mitophagy and cancer therapy. Cancers.

[104] Visentin B., Vekich J.A., Sibbald B.J., Cavalli A.L., Moreno K.M., Matteo R.G., Garland W.A., Lu Y., Yu S., Hall H.S. (2006). Validation of an anti-sphingosine-1-phosphate antibody as a potential therapeutic in reducing growth, invasion, and angiogenesis in multiple tumor lineages. Cancer Cell.

[105] Chan W.K., Lee H.M., Lee T.H., Kang C., Yong S.G. (2002). Extracellular membrane vesicles from tumor cells promote angiogenesis via sphingomyelin. Cancer Res..

[106] Riboni L., Viani P., Bassi R., Giussani P., Tettamanti G. (2001). Basic fibroblast growth factor-induced proliferation of primary astrocytes. evidence for the involvement of sphingomyelin biosynthesis. J. Biol. Chem..

[107] Liu B., Xiao J., Dong M., Qiu Z., Jin J. (2020). Human alkaline ceramidase 2 promotes the growth, invasion, and migration of hepatocellular carcinoma cells via sphingomyelin phosphodiesterase acid-like 3B. Cancer Sci..

[108] Morad S.A., Cabot M.C. (2013). Ceramide-orchestrated signalling in cancer cells. Nat. Rev. Cancer.

[109] Zama K., Mitsutake S., Okazaki T., Igarashi Y. (2018). Sphingomyelin in microdomains of the plasma membrane regulates amino acid-stimulated mTOR signal activation. Cell Biol. Int..

[110] Jing F., Jing C., Dai X., Zhou G., Hong L. (2021). Sphingomyelin synthase 2 but not sphingomyelin synthase 1 is upregulated in ovarian cancer and involved in migration, growth and survival via different mechanisms. Am. J. Transl. Res..

[111] Don A.S., Lim X.Y., Couttas T.A. (2014). Re-configuration of sphingolipid metabolism by oncogenic transformation. Biomolecules.

[112] Taniguchi M., Ueda Y., Matsushita M., Nagaya S., Hashizume C., Arai K., Kabayama K., Fukase K., Watanabe K., Wardhani L.O. (2020). Deficiency of sphingomyelin synthase 2 prolongs survival by the inhibition of lymphoma infiltration through ICAM-1 reduction. FASEB J..

[113] Deng Y., Hu J.C., He S.H., Lou B., Ding T.B., Yang J.T., Mo M.G., Ye D.Y., Zhou L., Jiang X.C. (2021). Sphingomyelin synthase 2 facilitates M2-like macrophage polarization and tumor progression in a mouse model of triple-negative breast cancer. Acta Pharmacol. Sin..

[114] Chongsathidkiet P., Jackson C., Koyama S., Loebel F., Cui X., Farber S.H., Woroniecka K., Elsamadicy A.A., Dechant C.A., Kemeny H.R. (2018). Sequestration of T cells in bone marrow in the setting of glioblastoma and other intracranial tumors. Nat. Med..

[115] Montfort A., Bertrand F., Rochotte J., Gilhodes J., Filleron T., Milhes J., Dufau C., Imbert C., Riond J., Tosolini M. (2021). Neutral sphingomyelinase 2 heightens anti-melanoma immune responses and anti-pd-1 therapy efficacy. Cancer Immunol. Res..

[116] Wang J., Li J., Gu J., Yu J., Guo S., Zhu Y., Ye D. (2015). Abnormal methylation status of FBXW10 and SMPD3, and associations with clinical characteristics in clear cell renal cell carcinoma. Oncol. Lett..

[117] Jabalee J., Towle R., Lawson J., Dickman C., Garnis C. (2020). Sphingomyelin phosphodiesterase 3 methylation and silencing in oral squamous cell carcinoma results in increased migration and invasion and altered stress response. Oncotarget.

[118] Tsuruo T. (1994). Mechanism of multidrug resistance and implication for therapy. Pathophysiology.

[119] Peetla C., Vijayaraghavalu S., Labhasetwar V. (2013). Biophysics of cell membrane lipids in cancer drug resistance: Implications for drug transport and drug delivery with nanoparticles. Adv. Drug Deliv. Rev..

[120] Emma B., Gfn B., Fms B., Asc B., Bs B., Rcf A., Ono B. (2020). Role of sphingomyelin on the interaction of the anticancer drug gemcitabine hydrochloride with cell membrane models. Colloids Surf. B.

[121] Xu J.X., Morii E., Liu Y., Nakamichi N., Ikeda J., Kimura H., Aozasa K. (2007). High tolerance to apoptotic stimuli induced by serum depletion and ceramide in side-population cells: High expression of CD55 as a novel character for side-population. Exp. Cell Res..

[122] Cervia D., Assi E., De Palma C., Giovarelli M., Bizzozero L., Pambianco S., Di Renzo I., Zecchini S., Moscheni C., Vantaggiato C. (2016). Essential role for acid sphingomyelinase-inhibited autophagy in melanoma response to cisplatin. Oncotarget.

[123] Grammatikos G., Teichgraber V., Carpinteiro A., Trarbach T., Weller M., Hengge U.R., Gulbins E. (2007). Overexpression of acid sphingomyelinase sensitizes glioma cells to chemotherapy. Antioxid. Redox. Signal..

[124] Lacour S., Hammann A., Grazide S., Lagadic-Gossmann D., Athias A., Sergent O., Laurent G., Gambert P., Solary E., Dimanche-Boitrel M.-T. (2004). Cisplatin-induced CD95 redistribution into membrane lipid rafts of HT29 human colon cancer cells. Cancer Res..

[125] Maurmann L., Belkacemi L., Adams N.R., Majmudar P.M., Moghaddas S., Bose R.N. (2015). A novel cisplatin mediated apoptosis pathway is associated with acid sphingomyelinase and FAS proapoptotic protein activation in ovarian cancer. Apoptosis.

[126] Smith E.L., Schuchman E.H. (2008). Acid sphingomyelinase overexpression enhances the antineoplastic effects of irradiation in vitro and in vivo. Mol. Ther..

[127] Zhong L., Li Y., Xiong L., Wang W., Wu M., Yuan T., Yang W., Tian C., Miao Z., Wang T. (2021). Small molecules in targeted cancer therapy: Advances, challenges, and future perspectives. Signal Transduct. Target. Ther..

[128] Phillips M.A., Gran M.L., Peppas N.A. (2010). Targeted nanodelivery of drugs and diagnostics. Nano Today.

[129] Wang Z., Little N., Chen J., Lambesis K.T., Le K.T., Han W., Scott A.J., Lu J. (2021). Immunogenic camptothesome nanovesicles comprising sphingomyelin-derived camptothecin bilayers for safe and synergistic cancer immunochemotherapy. Nat. Nanotechnol..

[130] Bouzo B.L., Lores S., Jatal R., Alijas S., Alonso M.J., Conejos-Sanchez I., de la Fuente M. (2021). Sphingomyelin nanosystems loaded with uroguanylin and etoposide for treating metastatic colorectal cancer. Sci. Rep..

[131] Medina O.P., Tower R.J., Medina T.P., Ashkenani F., Appold L., Bötcher M., Huber L., Will O., Ling Q., Hauser C. (2020). Multimodal targeted nanoparticle-based delivery system for pancreatic tumor imaging in cellular and animal models. Curr. Pharm. Des..

[132] Penate Medina T., Gerle M., Humbert J., Chu H., Kopnick A.L., Barkmann R., Garamus V.M., Sanz B., Purcz N., Will O. (2020). Lipid-iron nanoparticle with a cell stress release mechanism combined with a local alternating magnetic field enables site-activated drug release. Cancers.

[133] Masoumi F., Saraiva S.M., Bouzo B.L., Lopez-Lopez R., Esteller M., Diaz-Lagares A., de la Fuente M. (2021). Modulation of colorectal tumor behavior via lncrna tp53tg1-lipidic nanosystem. Pharmaceutics.

[134] Nagachinta S., Bouzo B.L., Vazquez-Rios A.J., Lopez R., Fuente M. (2020). Sphingomyelin-based nanosystems (sns) for the development of anticancer miRNA therapeutics. Pharmaceutics.

[135] Mutter N.L., Volaric J., Szymanski W., Feringa B.L., Maglia G. (2019). Reversible photocontrolled nanopore assembly. J. Am. Chem. Soc..

[136] Hassan A.H.E., Park H.R., Yoon Y.M., Kim H.I., Yoo S.Y., Lee K.W., Lee Y.S. (2019). Antiproliferative 3-deoxysphingomyelin analogs: Design, synthesis, biological evaluation and molecular docking of pyrrolidine-based 3-deoxysphingomyelin analogs as anticancer agents. Bioorganic Chem..

[137] Morad S.A., Messner M.C., Levin J.C., Abdelmageed N., Park H., Merrill A.H., Cabot M.C. (2013). Potential role of acid ceramidase in conversion of cytostatic to cytotoxic end-point in pancreatic cancer cells. Cancer Chemoth. Pharm..

[138] Flowers M., Fabrias G., Delgado A., Casas J., Abad J.L., Cabot M.C. (2012). C6-ceramide and targeted inhibition of acid ceramidase induce synergistic decreases in breast cancer cell growth. Breast Cancer Res. Treat..

[139] Huang H., Zhang Y., Liu X., Li Z., Xu W., He S., Huang Y., Zhang H. (2011). Acid sphingomyelinase contributes to evodiamine-induced apoptosis in human gastric cancer SGC-7901 cells. DNA Cell Biol..

[140] Kester M., Heakal Y., Fox T., Sharma A., Robertson G.P., Morgan T.T., Altinoglu E.I., Tabakovic A., Parette M.R., Rouse S.M. (2008). Calcium phosphate nanocomposite particles for in vitro imaging and encapsulated chemotherapeutic drug delivery to cancer cells. Nano Lett..

[141] Ganta S., Singh A., Patel N.R., Cacaccio J., Rawal Y.H., Davis B.J., Amiji M.M., Coleman T.P. (2014). Development of EGFR-targeted nanoemulsion for imaging and novel platinum therapy of ovarian cancer. Pharm. Res..

[142] Stover T., Kester M. (2003). Liposomal delivery enhances short-chain ceramide-induced apoptosis of breast cancer cells. J. Pharmacol. Exp. Ther..

[143] Chen L., Alrbyawi H., Poudel I., Arnold R.D., Babu R.J. (2019). Co-delivery of doxorubicin and ceramide in a liposomal formulation enhances cytotoxicity in murine B16Bl6 melanoma cell lines. AAPS PharmSciTech.

[144] Li G., Liu D., Kimchi E.T., Kaifi J.T., Qi X., Manjunath Y., Liu X., Deering T., Avella D.M., Fox T. (2018). Nanoliposome C6-ceramide increases the anti-tumor immune response and slows growth of liver tumors in mice. Gastroenterology.

[145] Teng Y., Li J., Hui S. (2010). C6 ceramide potentiates curcumin-induced cell death and apoptosis in melanoma cell lines in vitro. Cancer Chemother. Pharmacol..

[146] Jiang Y., DiVittore N.A., Kaiser J.M., Shanmugavelandy S.S., Fritz J.L., Heakal Y., Tagaram H.R., Cheng H., Cabot M.C., Staveley-O’Carroll K.F. (2011). Combinatorial therapies improve the therapeutic efficacy of nanoliposomal ceramide for pancreatic cancer. Cancer Biol. Ther..

[147] Pal S., Medatwal N., Kumar S., Kar A., Komalla V., Yavvari P.S., Mishra D., Rizvi Z.A., Nandan S., Malakar D. (2019). A localized chimeric hydrogel therapy combats tumor progression through alteration of sphingolipid metabolism. ACS Cent. Sci..

[148] Medatwal N., Ansari M.N., Kumar S., Pal S., Jha S.K., Verma P., Rana K., Dasgupta U., Bajaj A. (2020). Hydrogel-mediated delivery of celastrol and doxorubicin induces a synergistic effect on tumor regression via upregulation of ceramides. Nanoscale.

[149] Bi J., Khan A., Tang J., Armando A.M., Wu S., Zhang W., Gimple R.C., Reed A., Jing H., Koga T. (2021). Targeting glioblastoma signaling and metabolism with a re-purposed brain-penetrant drug. Cell. Rep..

[150] Liu Y., Wang X., Li J., Tang J., Li B., Zhang Y., Gu N., Yang F. (2021). Sphingosine 1-phosphate liposomes for targeted nitric oxide delivery to mediate anticancer effects against brain glioma tumors. Adv. Mater..

[151] Saddoughi S.A., Gencer S., Peterson Y.K., Ward K.E., Mukhopadhyay A., Oaks J., Bielawski J., Szulc Z.M., Thomas R.J., Selvam S.P. (2013). Sphingosine analogue drug FTY720 targets I2PP2A/SET and mediates lung tumour suppression via activation of PP2A-RIPK1-dependent necroptosis. EMBO Mol. Med..

[152] Companioni O., Mir C., Garcia-Mayea Y., ME L.L. (2021). Targeting Sphingolipids for Cancer Therapy. Front. Oncol..

[153] Meng Q., Zhao B., Xu Q., Xu X., Deng G., Li C., Luan L., Ren F., Wang H., Xu H. (2012). Indole-propionic acid derivatives as potent, S1P3-sparing and EAE efficacious sphingosine-1-phosphate 1 (S1P1) receptor agonists. Bioorg. Med. Chem. Lett..

[154] Luo D., Guo Z., Zhao X., Wu L., Liu X., Zhang Y., Zhang Y., Deng Z., Qu X., Cui S. (2021). Novel 5-fluorouracil sensitizers for colorectal cancer therapy: Design and synthesis of S1P receptor 2 (S1PR2) antagonists. Eur. J. Med. Chem..

[155] Beljanski V., Knaak C., Smith C.D. (2010). A novel sphingosine kinase inhibitor induces autophagy in tumor cells. J. Pharmacol. Exp. Ther..

[156] Huang X., Taeb S., Jahangiri S., Emmenegger U., Tran E., Bruce J., Mesci A., Korpela E., Vesprini D., Wong C.S. (2013). miRNA-95 mediates radioresistance in tumors by targeting the sphingolipid phosphatase SGPP1. Cancer Res..

[157] Billich A., Bornancin F., Mechtcheriakova D., Natt F., Huesken D., Baumruker T. (2005). Basal and induced sphingosine kinase 1 activity in A549 carcinoma cells: Function in cell survival and IL-1beta and TNF-alpha induced production of inflammatory mediators. Cell. Signal..

[158] Leroux M.E., Auzenne E., Evans R., Hail N., Spohn W., Ghosh S.C., Farquhar D., McDonnell T., Klostergaard J. (2007). Sphingolipids and the sphingosine kinase inhibitor, SKI II, induce BCL-2-independent apoptosis in human prostatic adenocarcinoma cells. Prostate.

[159] Liu H., Kong Y., Liu Z., Guo X., Yang B., Yin T., He H., Gou J., Zhang Y., Tang X. (2022). Sphingomyelin-based PEGylation Cu (DDC)(2) liposomes prepared via the dual function of Cu(2+) for cancer therapy: Facilitating DDC loading and exerting synergistic antitumor effects. Int. J. Pharm..

[160] Bidan N., Lores S., Vanhecke A., Nicolas V., Domenichini S., Lopez R., de la Fuente M., Mura S. (2022). Before in vivo studies: In vitro screening of sphingomyelin nanosystems using a relevant 3D multicellular pancreatic tumor spheroid model. Int. J. Pharm..

[161] Massiot J., Rosilio V., Ibrahim N., Yamamoto A., Nicolas V., Konovalov O., Tanaka M., Makky A. (2018). Newly synthesized lipid-porphyrin conjugates: Evaluation of their self-assembling properties, their miscibility with phospholipids and their photodynamic activity in vitro. Chemistry.

[162] Lim E.B., Haam S., Lee S.W. (2021). Sphingomyelin-based liposomes with different cholesterol contents and polydopamine coating as a controlled delivery system. Colloid Surface A.

[163] Zembruski N.C., Nguyen C.D., Theile D., Ali R.M., Herzog M., Hofhaus G., Heintz U., Burhenne J., Haefeli W.E., Weiss J. (2013). Liposomal sphingomyelin influences the cellular lipid profile of human lymphoblastic leukemia cells without effect on P-glycoprotein activity. Mol. Pharm..

[164] Zhang X., Kitatani K., Toyoshima M., Ishibashi M., Usui T., Minato J., Egiz M., Shigeta S., Fox T., Deering T. (2018). Ceramide nanoliposomes as a MLKL-dependent, necroptosis-inducing, chemotherapeutic reagent in ovarian cancer. Mol. Cancer Ther..

